# Louisville Medical News

**Published:** 1883-08

**Authors:** 


### LOUISVILLE MEDICAL NEWS.

We see it announced that Dr. L. S. McMurtry, one of the associate editors of the above journal, has retired. He will be succeeded by Dr. H. A. Cottell, formerly an editor of the same journal. We wish for the *News* the success it deserves under its able editorial staff.

